# A Continuum Deformation Approach for Growth Analysis of COVID-19 in the United States

**DOI:** 10.1038/s41598-021-97021-z

**Published:** 2021-09-08

**Authors:** Sadra Hemmati, Hossein Rastgoftar

**Affiliations:** 1 Mechanical Engineering Department, Villanova University, Pennsylvania, 19085 USA; 2 Aerospace and Mechanical Engineering Department, University of Arizona, Arizona, 85721 USA

**Keywords:** Viral infection, Applied mathematics

## Abstract

The COVID-19 global pandemic has significantly impacted every aspect of life all over the world. The United States is reported to have suffered more than 20% of the global casualties from this pandemic. It is imperative to investigate the growth dynamics of the disease in the US based on varying geographical and governmental factors that best manifest itself in each State of the Country. This paper utilizes a hybrid machine learning and continuum deformation-based approach for analyzing the stability and growth rate of the pandemic. To this end, principal stress values of the pandemic continuum body are obtained using Mohr’s Circle method and overlapping, moving windows of data are analysed successively. This helps in finding the correlations between the growth rate and Governments’ action/Public’s reaction. Government actions include “state of emergency”, “shelter at place”, and “phase declarations”. We also consider the vaccination rate milestones, which shows us the coordinated Governments’ action/Public’s reaction. Finally, a number of recommendations are made to the Governments and people for better management of future pandemics.

## Introduction

The first death caused by COVID-19 in the United States is believed to have occurred in Santa Clara County, California on the February, 6th, and the virus has rapidly grown across the country since then. Studies have shown that the virus is dominantly transmitted through close contact with infected people and contaminated surfaces as well as respiratory droplets^1,2^. This spread includes a dispersion dynamics, and trajectory tracking techniques in the field of Control Theory can be effectively utilized.

Usually, the daily reports of pandemic statistics only include the deaths, total cases, active cases, and other similar explicit demographic parameters for varying geographical locations. Thus, models that can learn well from these types of data sets to infer the spread dynamics are very valuable and versatile for State-level decision making. Considering the different scales of analysis (global, between two countries, inside countries, between all States of a country, between Counties of a State, etc), obtaining detailed information regarding factors that affect transmission of the disease are challenging. This paper offers a new continuum-mechanics-based model to analyze the growth of the pandemic diseases and evaluate the effectiveness of the non-pharmaceutical and pharmaceutical actions. We treat evolution of a pandemic disease as a continuum deformation problem in three dimensional *T*–*D*–*R* space where *T*, *D*, and *R* denote the total number of infected cases, the total number of the deaths, and the total number of recoveries, respectively. We focus on the growth of COVID-19 in the US States and the District of Columbia. Hence, the pandemic continuum consists of 51 particles of 3-D deformable bodies evolving in the *T*–*D*–*R* space.

**Figure 1:**
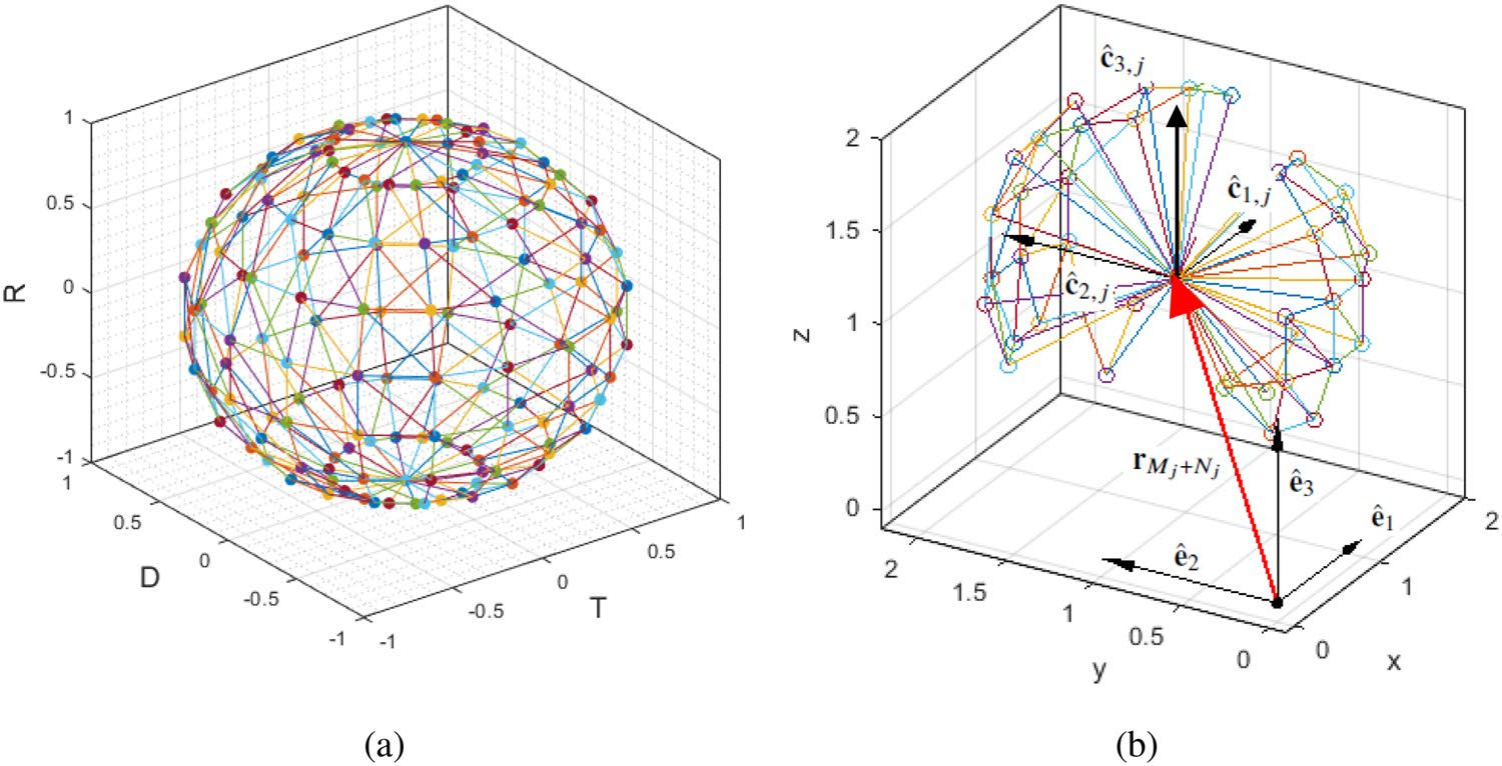
(**a**) *T*–*D*–*R* space polyhedralization for $j \in {\mathcal {C}}$, using the Eqs. (9a) and (9b). The polytopes are contained in a sphere of radius 1 at the initial moment, and conserve their orientation and are deformed and elongated during the pandemic growth. (**b**) The containment polytope $1\in {\mathcal {C}}$ at day $k= 100$. To determine the containment polytope we choose $p=15$ and $q=27$, therefore, ${\mathcal {B}}_1=\left\{ 1,2,\ldots ,N-1\right\}$ and ${\mathcal {I}}_1=\{N\}$. The origin of the local coordinate of polytope $1\in {\mathcal {C}}$ is positioned at $\bar{{\mathbf {r}}}_{N,g}=1.1 \times 10^4\hat{{\mathbf {e}}}_1+1.3 \times 10^4\hat{{\mathbf {e}}}_2+1.2 \times 10^4\hat{{\mathbf {e}}}_3$, therefore $\mu _{T,1}=1.1 \times 10^4$, $\mu _{D,1}=1.3 \times 10^4$, and $\mu _{R,1}=1.2 \times 10^4$ (See Remark 1).

### Related work

Different types of models with varying theoretical principles have been used for pandemic growth prediction and stability analysis. The methods range form statistical inference and correlation, to differential equation-based models. SIR method^3–5^, SEIR dynamics^6–10^, Metapopulation Dynamics^11,12^, Mean-Field Theory^13,14^, Bayesian regression^2^, maximum likelihood^15^, mass-conservation based^16^ are examples of existing models used to estimate the dynamics of infectious diseases.

In^17^, the authors, using the Moving Regression (MR) technique and a Hidden Markov Model (HMM), aimed at developing a simple framework for prediction of the growth rate (cases/day) and growth acceleration (cases/day$^2$) of COVID-19 cases in real-time and study the effects of public health measures on the prevalence of COVID-19. Another correlation study aimed at studying the seasonal temperature variations on pandemic growth. The authors report their developed model can explain 36% of the variation in maximum COVID-19 growth rates based on weather and demography (17%) and country-specific effects (19%)^18^. In^19^, the authors model the US epidemic at the State-level, using publicly available death data within a Bayesian hierarchical semi-mechanistic framework in which the SARS-COV2 transmission was predicted using mobility trends. It is reported that Statewide stay-at-home orders had the strongest causal impact on reducing social interaction and mobility. For instance, in^20^, the authors report that the Statewide stay-at-home orders result in a steady decline in confirmed cases, starting from ten days after implementation and reaching a 37% decrease after fifteen days, consistent with the testing practices and incubation period of the disease. They mention this executive order had the strongest causal impact on reducing social interactions. Therefore, we examine our results with the stay-at-home executive orders of each State.

### Contributions and outline

The main objective of this paper is to analyze the growth of the pandemic disease by analyzing deformation of the pandemic continua in the *T*–*D*–*R* space. To this end, we first apply the k-means algorithm, divide the US States/district into a finite number of clusters and determine the centroid of each cluster in the *T*–*D*–*R* space. We then offer a novel polyhedral learning approach to contain each cluster by a 3-D polytope. Compared to the existing research and the authors’ previous work, this paper offers the following novel contributions:

1. The existing learning methods solve a nonlinear optimization problem to determine the solution of a classification problem. Therefore, the solution of a classification problem may not necessarily converge to the global optimum. However, the proposed polyhedral learning does not deal with the convergence issue of the existing approaches since it determines the boundary of the containment polytopes by assigning maxima of finite sets of discrete variables.
2. The proposed polyhedral learning method ensures that the training data are all enclosed by the containment polytopes.
3. To the best of our knowledge, this is the first paper that models evolution of a pandemic disease as a continuum deformation coordination.

In this paper, we integrate model and data to analyze the pandemic growth; investigate the effectiveness of the nationwide/state action; and evaluate public reaction to the stability of the pandemic evolution in the US. This paper is organized as follows: A polyhedralization method is developed in “Motion space polyhedralization” and followed by the polyhedral learning in “Polyhedral learning of a pandemic disease”. Pandemic disease evolution is modeled as continuum deformation in “Pandemic disease evolution”. Results of stability analysis of pandemic evolution is discussed in “Growth analysis and pandemic stages”. The conclusion is presented in “Conclusion”.

## Motion space polyhedralization

The pandemic grows in a 3-D space with coordinates *T*, *D*, and *R* (previously defined) while the points in the space are clustered into *m* groups. The evolving clusters are then contained by *m* deformable polytopes in the *T*–*D*–*R* space where identification numbers of the containment polytopes are defined by set ${\mathcal {C}}=\left\{ 1,2,\ldots ,m\right\}$. Configuration of every polytope $j\in {\mathcal {C}}$ is determined by *N* characteristic nodes and formed by $\rho$ tetrahedrons as described below.

### Characteristic nodes of polytope $j\in {\mathcal {C}}$

The geometry and location of polytope $j\in {\mathcal {C}}$ is assigned by *N* nodes in the *T*–*D*–*R* space that are identified by set

1 $$\begin{aligned} {\mathcal {L}}_j=\left\{ \left( j-1\right) N+1,\ldots ,jN\right\} . \end{aligned}$$

Set ${\mathcal {L}}_j$ can be expressed as

2 $$\begin{aligned} {\mathcal {L}}_j={\mathcal {B}}_j\bigcup {\mathcal {I}}_j,\qquad \forall j\in {\mathcal {C}}, \end{aligned}$$

where singleton ${\mathcal {I}}_j=\left\{ jN\right\}$ defines the identification number of the interior characteristic node of polytope $j\in {\mathcal {C}}$ and ${\mathcal {B}}_j={\mathcal {L}}_j\setminus {\mathcal {I}}_j$ defines identification numbers of the boundary nodes of polytope $j\in {\mathcal {C}}$.

**Figure 2:**
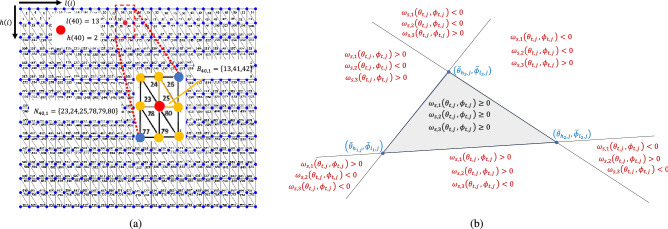
(**a**) The indexing method used to provide reference for each boundary node $i\in {{\mathcal {B}}}_{j}$ and $j \in {\mathcal {C}}$ shown in Fig. 1a. (**b**) Discretization of the $\theta -\phi$ plane based on the signs of components of vector $\varvec{\Omega }_s\left( \theta _{t,j},\phi _{t,j}\right)$ introduced in Eq. (20).

#### Local coordinates of characteristic nodes

Every index number $i\in {\mathcal {B}}_{j}$ can be converted to unique $l\in \{1,\ldots ,p\}$ and $h\in \{1,\ldots ,q\}$ coordinates for every $j\in {\mathcal {C}}$. More specifically, $l:{\mathcal {B}}_j\rightarrow \{1,\ldots ,p \}$ and $h:{\mathcal {B}}_j\rightarrow \{1,\ldots ,q \}$ are defined as follows:

3a $$\begin{aligned} \forall j\in {\mathcal {C}}, i\in {\mathcal {B}}_j,\qquad l(i)={\left\{ \begin{array}{ll} \left\lfloor {i\over q}\right\rfloor +1&{}{\text{ If } }i-q\left\lfloor {i\over q}\right\rfloor \ne 0\\ \left\lfloor {i\over q}\right\rfloor &{}{\text {If}}\;i-q\left\lfloor {i\over q}\right\rfloor = 0\\ \end{array}\right. }, \end{aligned}$$

3b $$\begin{aligned} j\in {\mathcal {C}},~i\in {\mathcal {B}}_j,\qquad h(i)={\left\{ \begin{array}{ll} i-q\left\lfloor {i\over q}\right\rfloor &{}{\text {If }}i-q\left\lfloor {i\over q}\right\rfloor \ne 0\\ q&{}{\text {If }}i-q\left\lfloor {i\over q}\right\rfloor = 0\\ \end{array}\right. } . \end{aligned}$$

On the other hand, $i\in {\mathcal {B}}_j$ can be defined based on positive integers $l\in \left\{ 1,\ldots ,p\right\}$ and $h\in \left\{ 1,\ldots ,q\right\}$ by

4 $$\begin{aligned} i\left( l,h\right) =\left( l-1\right) q+h,\qquad \forall j\in {\mathcal {C}}. \end{aligned}$$

#### Local and global positions of characteristic nodes

The global and local positions of node $i\in {\mathcal {L}}_j$ are denoted by $\bar{{\mathbf {r}}}_{i,g}$ and $\bar{{\mathbf {r}}}_{i,j,c}$, respectively. The global position is expressed with respect to the global coordinate system with fixed unit base vectors $\hat{{\mathbf {e}}}_1$, $\hat{{\mathbf {e}}}_2$, and $\hat{{\mathbf {e}}}_3$. Also, the local position of characteristic node $i\in {\mathcal {L}}_j$ is expressed with respect to local coordinate system of polytope *j* whose base vectors are denoted by $\hat{{\mathbf {c}}}_{1,j}$, $\hat{{\mathbf {c}}}_{2,j}$, and $\hat{{\mathbf {c}}}_{3,j}$. Note that the origin of the local coordinate system of polytope $j\in {\mathcal {C}}$ is located at $\bar{{\mathbf {r}}}_{jN,g}$, where $jN\in {\mathcal {I}}_{j}$ is the index number of the characteristic interior node of polytope $j\in {\mathcal {C}}$.

We assume that the local coordinate system of polytope $j\in {\mathcal {C}}$ translates with no rotation in the *T*–*D*–*R* space, thus $$\hat{{\mathbf {e}}}_1=\hat{{\mathbf {c}}}_{1,j}=\begin{bmatrix} 1&0&0 \end{bmatrix}^T$$, $$\hat{{\mathbf {e}}}_2=\hat{{\mathbf {c}}}_{2,j}=\begin{bmatrix} 0&1&0 \end{bmatrix}^T$$, $$\hat{{\mathbf {e}}}_3=\hat{{\mathbf {c}}}_{3,j}=\begin{bmatrix} 0&0&1 \end{bmatrix}^T$$ for every polytope $j\in {\mathcal {C}}$. As a result, $\bar{{\mathbf {r}}}_{i,g}={\bar{T}}_{i,g}\hat{{\mathbf {e}}}_1+{\bar{D}}_{i,g}\hat{{\mathbf {e}}}_2+{\bar{R}}_{i,g}\hat{{\mathbf {e}}}_3$ and $\bar{{\mathbf {r}}}_{i,j,c}={\bar{T}}_{i,j,c}\hat{{\mathbf {c}}}_{1,j}+{\bar{D}}_{i,j,c}\hat{{\mathbf {c}}}_{2,j}+{\bar{R}}_{i,j,c}\hat{{\mathbf {c}}}_{3,j}$ are related by

5 $$\begin{aligned} \bar{{\mathbf {r}}}_{i,g}(k)=\bar{{\mathbf {r}}}_{jN,g}(k)+\bar{{\mathbf {r}}}_{i,j,c}(k),\qquad \forall i\in {\mathcal {B}}_j,~\forall j\in {\mathcal {C}}, \end{aligned}$$

where *k* is the number of days from the date of establishment of a pandemic disease (see Fig. 1b). Per Eq. (5), components of the global and local positions are related by

6 $$\begin{aligned} {\left\{ \begin{array}{ll} {\bar{T}}_{i,g}(k)={\bar{T}}_{jN,g}(k)+{\bar{T}}_{i,j,c}(k)\\ {\bar{D}}_{i,g}(k)={\bar{D}}_{jN,g}(k)+{\bar{D}}_{i,j,c}(k)\\ {\bar{R}}_{i,g}(k)={\bar{R}}_{jN,g}(k)+{\bar{R}}_{i,j,c}(k)\\ \end{array}\right. } \qquad \forall i\in {\mathcal {B}}_j,~\forall j\in {\mathcal {C}}, \end{aligned}$$

at day *k*. We express the local position of node $i\in {\mathcal {B}}_j$ by

7 $$\begin{aligned} \bar{{\mathbf {r}}}_{i,j,c}={\bar{d}}_{i,j}\hat{{\mathbf {n}}}_{i,j,c}, \end{aligned}$$

where ${\bar{d}}_{i,j}$ is distance of boundary node $i\in {\mathcal {B}}_j$ from the characteristic interior node $jN\in {\mathcal {I}}_j$, and

8 $$\begin{aligned} \hat{{\mathbf {n}}}_{i,j,c}= \begin{bmatrix} \cos {\bar{\theta }}_{h,j}\cos {\bar{\phi }}_{l,j}&\sin {\bar{\theta }}_{h,j}\cos {\bar{\phi }}_{l,j}&\sin {\bar{\phi }}_{l,j} \end{bmatrix} ^T, \end{aligned}$$

with latitude and azimuth angles

9a $$\begin{aligned} {\bar{\theta }}_{h,j}= & {} {2\pi h\over q}, \end{aligned}$$

9b $$\begin{aligned} {\bar{\phi }}_{l,j}= & {} {\pi \left( l-1\right) \over p-1}, \end{aligned}$$

for $l\in \{1,\ldots ,p\}$ and $h\in \{1,\ldots ,q\}$. Thus, the direction unit vector $\hat{{\mathbf {n}}}_{i,j,c}$ is known for every boundary node $i\in {\mathcal {B}}_j$ and every cluster $j\in {\mathcal {C}}$.

##### *Remark 1*

In the continuation of this paper, position of the characteristic interior node of polytope $j\in {\mathcal {C}}$ is denoted by

10 $$\begin{aligned} \bar{{\mathbf {r}}}_{jN}=\mu _{T,j}\hat{{\mathbf {e}}}_1+\mu _{D,j}\hat{{\mathbf {e}}}_2+\mu _{R,j}\hat{{\mathbf {e}}}_3 \end{aligned}$$

and assigned using the K-means clustering algorithm in “K-means clustering algorithm”, i.e. $\mu _{T,j}=T_{jN,j}$, $\mu _{D,j}=D_{jN,j}$, and $\mu _{R,j}=R_{jN,j}$ for every $j\in {\mathcal {C}}$.

### Characteristic tetrahedrons of polytope $j\in {\mathcal {C}}$

The boundary of polytope $j\in {\mathcal {C}}$ consists of $\rho =2q\left( p-1\right)$ triangular cells, defined by set ${\mathcal {S}}_j=\left\{ 1,\ldots ,2q\left( p-1\right) \right\}$ (see Fig. 2a ). For every $j\in {\mathcal {C}}$, set ${\mathcal {S}}_j$ can be expressed by

11 $$\begin{aligned} {\mathcal {S}}_j={\mathcal {S}}_{j,o}\bigcup {\mathcal {S}}_{j,e}, \end{aligned}$$

where

$$\begin{aligned} S_{j,o}=\left\{ 2i\left( l,h\right) -1:l\in \left\{ 1,\cdots ,p-1\right\} ,h\in \left\{ 1,\ldots ,q \right\} \right\} \qquad {\text {and}}\qquad S_{j,e}=\left\{ 2i\left( l,h\right) :l\in \left\{ 1,\ldots ,p-1\right\} ,h\in \left\{ 1,\ldots ,q \right\} \right\} , \end{aligned}$$

define triangular cells with odd and even identification numbers, respectively, where *i*(*l*, *h*) is defined by Eq. (4) for given $l\in \{1,\ldots ,p-1\}$ and $h\in \{1,\ldots ,q\}$. Additionally, set ${\mathcal {B}}_j$ can be expressed by

12 $$\begin{aligned} {\mathcal {B}}_j=\bigcup _{s\in {\mathcal {S}}_j}{\mathcal {B}}_{j,s}=\bigcup _{l=1}^{p-1}\bigcup _{h=1}^{q}\left( {\mathcal {B}}_{j,2i\left( l,h\right) -1}\bigcup {\mathcal {B}}_{j,2i\left( l,h\right) }\right) ,\qquad \forall j\in {\mathcal {C}}, \end{aligned}$$

where

13a $$\begin{aligned} {\mathcal {B}}_{j,2i-1}= & {} {\left\{ \begin{array}{ll} \left\{ i,i+q,i+q+1\right\} &{}{\text {If }}h(i)<q\\ \left\{ i,i+q,i+1\right\} &{}{\text {If }}h(i)=q\\ \end{array}\right. }, \end{aligned}$$

13b $$\begin{aligned} {\mathcal {B}}_{j,2i}= & {} {\left\{ \begin{array}{ll} \left\{ i,i+q+1,i+q+1\right\} &{}{\text {If }}h(i)<q\\ \left\{ i,i+1,i-q+1\right\} &{}{\text {If }}h(i)=q\\ \end{array}\right. } . \end{aligned}$$

Note that ${\mathcal {B}}_{j,s}$ defines the identification numbers of the vertices of triangle $s\in {\mathcal {S}}_j$ on the boundary of polytope $j\in {\mathcal {C}}$. If $s\in {\mathcal {S}}_j$ is an odd number, Eq. (13a) defines vertices of triangular cell $s\in {\mathcal {S}}_j$. Otherwise, Eq. (13b) identifies vertices of triangle $s\in {\mathcal {S}}_j$.

For every node $i\in {\mathcal {B}}_j$ of polytope $j\in {\mathcal {C}}$, set

14 $$\begin{aligned} {\mathcal {N}}_{i,j}=\left\{ s\in {\mathcal {S}}_j:i\in {\mathcal {B}}_{j,s} \right\} , \end{aligned}$$

defines the index numbers of the triangular cells on the boundary of polytope $j\in {\mathcal {C}}$ sharing common node $i\in {\mathcal {B}}_j$.

Figure 2a illustrates the configurations of the triangular cells on the boundary of every containment polytope $j\in {\mathcal {C}}$ for $p=15$ and $q=27$. Figure 2a also shows the nodes of triangular cell $25\in {\mathcal {S}}_j$, defined by ${\mathcal {B}}_{j,25}$, and the triangular cells sharing the common boundary node $40\in {\mathcal {B}}_j$ and defined by set ${\mathcal {N}}_{40,j}$ for every $j\in {\mathcal {C}}$, at day $k= 100$. To determine the containment polytope, we choose $p=10$ and $q=18$. Therefore, ${\mathcal {B}}_1=\left\{ 1,2,\ldots ,N-1\right\}$ and ${\mathcal {I}}_1=\{N\}$, where *N* is 420 for this study. The origin of the local coordinate system of polytope $1\in {\mathcal {C}}$ is positioned at $\bar{{\mathbf {r}}}_{N,g}=1.1 \times 10^4\hat{{\mathbf {e}}}_1+1.3 \times 10^4\hat{{\mathbf {e}}}_2+1.2 \times 10^4\hat{{\mathbf {e}}}_3$, therefore $\mu _{T,1}=1.1 \times 10^4$, $\mu _{D,1}=1.3 \times 10^4$, and $\mu _{R,1}=1.2 \times 10^4$ (See Remark 1).

## Polyhedral learning of a pandemic disease

Let set ${\mathcal {F}}$, identifying a finite number of training data points, be expressed by

15 $$\begin{aligned} {\mathcal {F}}=\bigcup _{j\in {\mathcal {C}}}{\mathcal {F}}_j, \end{aligned}$$

where ${\mathcal {F}}_1$ through ${\mathcal {F}}_m$ are disjoint subsets of ${\mathcal {F}}$; set ${\mathcal {F}}_j=\left\{ 1,\ldots ,f_j\right\}$ defines the training data points belonging to class $j\in {\mathcal {C}}$. In this paper, set ${\mathcal {F}}$ defines a total of 51 triplet data points informing about the total number infected cases, deaths, and recoveries in 50 US States and the District of Colombia.

The data points provided by set ${\mathcal {F}}$ is used to determine the geometry of the containment polytopes deforming in the *T*–*D*–*R* space. To this end, we first apply the K-means algorithm to cluster the US States and Washington DC into *m* groups defined by ${\mathcal {F}}_1$ through ${\mathcal {F}}_m$ and to determine the centroids of clusters ${\mathcal {F}}_1$ through ${\mathcal {F}}_m$ denoted by $\bar{{\mathbf {r}}}_{jN}\in {\mathcal {I}}_j\subset {\mathcal {F}}_j$ for every $j\in {\mathcal {C}}=\left\{ 1,\ldots ,m\right\}$ (See Remark 1). Then, the data points belonging set ${\mathcal {F}}_j\setminus {\mathcal {I}}_j$ are used to determine the boundary of polytope $j\in {\mathcal {C}}$ via assigning local positions $\bar{{\mathbf {r}}}_{(j-1)N+1}$ through $\bar{{\mathbf {r}}}_{jN-1}$.

### K-means clustering algorithm

We utilize an unsupervised learning method to cluster the finite data points available by set ${\mathcal {F}}=\bigcup _{j\in {\mathcal {C}}}{\mathcal {F}}_j$, into $m=|{\mathcal {C}}|$ clusters, namely the K-means clustering, explained in Algorithm 1. This algorithm works by calculating the distances of each data point to the center of cluster *j*. This method consists of two steps:

1. **Cluster assignment step** In the first step of the Algorithm 1, for each cluster $j\in {\mathcal {C}}$, and $|{\mathcal {C}}|=m$, a random vector (with the size of number of clusters, *m*) is generated to initialize the learning. Using the distance metrics appropriate to the data dimensions, the boundaries cluster *j* sub-space is calculated.
2. **Center update step** In the second step of the Algorithm 1, the location of each centroid is updated using the newly generated boundaries from the step *i* such that the cluster centers are moved to the average of each cluster points.

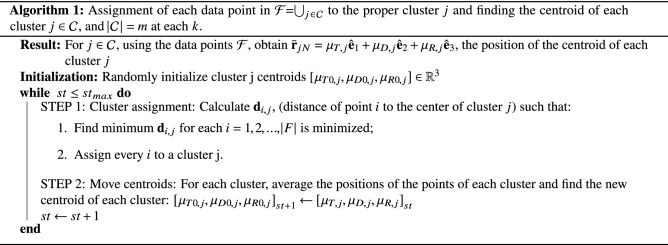

### Local position determination

We denote the global and local positions of training data $t\in {\mathcal {F}}_j$ by ${\mathbf {r}}_{t,g}$ and ${\mathbf {r}}_{t,j,c}$ for every $j\in {\mathcal {C}}$, where ${\mathbf {r}}_{t,g}=T_{t,g}\hat{{\mathbf {e}}}_1+D_{t,g}\hat{{\mathbf {e}}}_2+R_{t,g}\hat{{\mathbf {e}}}_3$ and ${\mathbf {r}}_{t,j,c}=T_{t,j,c}\hat{{\mathbf {c}}}_{1,j}+D_{t,j,c}\hat{{\mathbf {c}}}_{2,j}+R_{t,j,c}\hat{{\mathbf {c}}}_{3,j}$ are related by

16 $$\begin{aligned} \forall j\in {\mathcal {C}},~\forall t\in {\mathcal {F}}_j,\qquad {\mathbf {r}}_{t,j,c}={\mathbf {r}}_{t,g}-{\mathbf {r}}_{jN,g}. \end{aligned}$$

The local position of training data point $t\in {\mathcal {F}}_j$ ($j\in {\mathcal {C}}$) is expressed as follows:

17 $$\begin{aligned} {\mathbf {r}}_{t,j,g}={d}_{t,j,c}\begin{bmatrix} \cos \theta _{t,j}\sin \phi _{t,j}\\ \sin \theta _{t,j}\sin \phi _{t,j}\\ \cos \phi _{t,j}\\ \end{bmatrix} , \end{aligned}$$

where

$$\begin{aligned} d_{t,j}=\sqrt{T_{t,j,c}^2+D_{t,j,c}^2+R_{t,j,c}^2},\qquad \theta _{t,j}=\tan ^{-1}\left( {D_{t,j,c}\over T_{t,j,c}}\right) ,\qquad \phi _{t,j}=\cos ^{-1}\left( {R_{t,j,c}\over \sqrt{T_{t,j,c}^2+D_{t,j,c}^2+R_{t,j,c}^2}}\right) . \end{aligned}$$

For every polytope $j\in {\mathcal {C}}$, we determine the smallest polytope containing all training data points defined by set ${\mathcal {F}}_j\setminus {\mathcal {I}}_j$ by assigning the phase angle and radial distance of every training data as described below.

#### Step 1: Phase assignment

Let $\beta _{j,s}=\left\{ s_1,s_2,s_3\right\}$ define the vertices of triangular cell $s\in {\mathcal {S}}_j$ on the boundary of polytope $j\in {\mathcal {C}}$. Given index numbers $s_1\in {\mathcal {B}}_j$, $s_2\in {\mathcal {B}}_j$, and $s_3\in {\mathcal {B}}_j$, we can use Eqs. (3a) and (3b) to obtain $l_k=l\left( s_k \right) \in \left\{ 1,\ldots ,p\right\}$ and $h_k=h\left( s_k \right) \in \left\{ 1,\ldots ,q\right\}$ for $k=1,2,3$. By invoking Eq. (7), we can write

18 $$\begin{aligned} \bar{{\mathbf {r}}}_{s_k,j,c}={\bar{d}}_{s_k,j} \bar{{\mathbf {n}}}_{s_k,j,c}, \end{aligned}$$

where

19 $$\begin{aligned} \bar{{\mathbf {n}}}_{s_k,j,c}=\begin{bmatrix} \cos \theta _{h_k,j}\cos \phi _{l_k,j}\\ \sin \theta _{h_k,j}\cos \phi _{l_k,j}\\ \sin \phi _{l_k,j}\\ \end{bmatrix} \end{aligned}$$

for $k=1,2,3$ where $s_k\in {\mathcal {B}}_{j,s}$ $s\in {\mathcal {S}}_j$, and $j\in {\mathcal {C}}$. We define vector function

20 $$\begin{aligned} \varvec{\Omega }_s\left( \theta _{t,j},\phi _{t,j}\right) =\begin{bmatrix} \omega _{s,1}\left( \theta _{t,j},\phi _{t,j}\right) \\ \omega _{s,2}\left( \theta _{t,j},\phi _{t,j}\right) \\ \omega _{s,3}\left( \theta _{t,j},\phi _{t,j}\right) \end{bmatrix}= \begin{bmatrix} {\bar{\theta }}_{h_1,j}&{}{\bar{\theta }}_{h_2,j}&{}{\bar{\theta }}_{h_3,j}\\ {\bar{\phi }}_{l_1,j}&{}{\bar{\phi }}_{l_2,j}&{}{\bar{\phi }}_{l_3,j}\\ 1&{}1&{}1 \end{bmatrix} ^{-1} \begin{bmatrix} \theta _{t,j}\\ \phi _{t,j}\\ 1 \end{bmatrix} \end{aligned}$$

to determine the phase angle of the training data points define by set ${\mathcal {F}}$ according the following rules:

- If $\varvec{\Omega }_s\left( \theta _{t,j},\phi _{t,j}\right) \ge {\mathbf {0}}$, then, $\left( \theta _{t,j},\phi _{t,j}\right)$ is inside the triangle with vertices $\left( {\bar{\theta }}_{h_1,j},{\bar{\phi }}_{l_1,j}\right)$, $\left( {\bar{\theta }}_{h_2,j},{\bar{\phi }}_{l_2,j}\right)$, and $\left( {\bar{\theta }}_{h_3,j},{\bar{\phi }}_{l_3,j}\right)$ (see Fig. 2b) .
- If $\omega _{s,1}\left( \theta _{t,j},\phi _{t,j}\right)$, $\omega _{s,2}\left( \theta _{t,j},\phi _{t,j}\right)$ $\omega _{s,3}\left( \theta _{t,j},\phi _{t,j}\right)$ are not all non-negative, then, $\left( \theta _{t,j},\phi _{t,j}\right)$ is outside the triangle with vertices $\left( {\bar{\theta }}_{h_1,j},{\bar{\phi }}_{l_1,j}\right)$, $\left( {\bar{\theta }}_{h_2,j},{\bar{\phi }}_{l_2,j}\right)$, and $\left( {\bar{\theta }}_{h_3,j},{\bar{\phi }}_{l_3,j}\right)$

##### *Remark 2*

By using vector function $\varvec{\Omega }_s\left( \theta _{t,j},\phi _{t,j}\right)$, we can express set

21 $$\begin{aligned} \forall j\in {\mathcal {C}},\qquad {\mathcal {F}}_{j}=\bigcup _{s\in {\mathcal {S}}_j}\hat{{\mathcal {F}}}_{j,s} \end{aligned}$$

where $\hat{{\mathcal {F}}}_{j,1}$, $\cdots$, $\hat{{\mathcal {F}}}_{j,\rho }$ are disjoint subsets of set ${\mathcal {F}}_j$, and

22 $$\begin{aligned} \hat{{\mathcal {F}}}_{j,s}=\left\{ t\in {\mathcal {F}}_j:\varvec{\Omega }_s\left( \theta _{t,j},\phi _{t,j}\right) \ge {\mathbf {0}} \right\}, \qquad \forall j\in {\mathcal {C}},~\forall s\in {\mathcal {S}}_j=\left\{ 1,\cdots ,\rho \right\} . \end{aligned}$$

#### Step 2: Assignment of radial distances of boundary nodes

Let the training data set ${\mathcal {F}}_j$ be expressed by

23 $$\begin{aligned} \qquad {\mathcal {F}}_j=\bigcup _{i\in {\mathcal {B}}_j}\tilde{{\mathcal {F}}}_{j,i}=\bigcup _{i\in {\mathcal {B}}_j}\bigcup _{s\in {\mathcal {N}}_{i,j}}\hat{{\mathcal {F}}}_{j,s},\qquad j\in {\mathcal {C}}, \end{aligned}$$

where $\tilde{{\mathcal {F}}}_{j,i}$ defines all training data points that are enclosed by the tetrahedrons sharing the common node $i\in {\mathcal {B}}_j$ on the boundary of polytope $j\in {\mathcal {C}}$. The polytope $j\in {\mathcal {C}}$ encloses all training points, defined by set ${\mathcal {F}}_j$, if

24 $$\begin{aligned} j\in {\mathcal {C}},\qquad {\bar{d}}_{i,j}=\mathop {\hbox {argmax}}\limits _{t\in \tilde{{\mathcal {F}}}_{j,i}}\dfrac{1}{{\mathbf {r}}_{t,c,j}\cdot \hat{{\mathbf {n}}}_{i,j,c}} \end{aligned}$$

where $\hat{{\mathbf {n}}}_{i,j,c}$ is defined by (8) and “$\cdot$” is the dot product symbol.

## Pandemic disease evolution

Evolution of polytope $j\in {\mathcal {C}}$ in the *T*–*D*–*R* space is defined by

25 $$\begin{aligned} {\mathbf {x}}_{s,j}\left( k\right) ={\mathbf {Q}}_{s,j}\left( k\right) {\mathbf {x}}_{s,j,0}+{\mathbf {f}}_{s,j}(k) \end{aligned}$$

for every tetrahedron $s\in {\mathcal {S}}_j$ at discrete time $k=1,2,\ldots$, where *k* denotes the number of days from the establishment of a pandemic disease. For every $s\in {\mathcal {S}}_j$ and $j\in {\mathcal {C}}$,

$$\begin{aligned} {\mathbf {Q}}_{s,j}(k)=\begin{bmatrix} Q_{1,1,s,j}(k)&{}Q_{1,2,s,j}(k)&{}Q_{1,3,s,j}(k)\\ Q_{2,1,s,j}(k)&{}Q_{2,2,s,j}(k)&{}Q_{2,3,s,j}(k)\\ Q_{3,1,s,j}(k)&{}Q_{3,2,s,j}(k)&{}Q_{3,3,s,j}(k)\\ \end{bmatrix}\in {\mathbb {R}}^{3\times 3}\qquad {\text {and}}\qquad {\mathbf {f}}_{s,j}(k)=\begin{bmatrix} f_{1,s,j}(k)&f_{2,s,j}(k)&f_{3,s,j}(k) \end{bmatrix}\in {\mathbb {R}}^{3\times 1} \end{aligned}$$

are non-singular Jacobian matrix and the rigid-body displacement vector, respectively.

Note that $${\mathbf {x}}_{s,j,0}=\begin{bmatrix} T_{s,j,0}/100&D_{s,j,0}&R_{s,j,0}/100 \end{bmatrix} ^T\in {\mathbb {R}}^{3\times 1}$$ is the reference position of interior point of tetrahedron $s\in {\mathcal {S}}_j$ of polytope $j\in {\mathcal {C}}$ that is mapped to $${\mathbf {x}}_{s,j}(k)={\mathbf {x}}_{s,j}(k)=\begin{bmatrix} T_{s,j}(k)/100&D_{s,j}(k)&R_{s,j}(k)/100&\end{bmatrix} ^T\in {\mathbb {R}}^{3\times 1}$$ at day $k=1,2,\ldots$. The division of *T* and *R* by a scaling factor (100) is performed to regularize the order of magnitude of the elements of the ${\mathbf {x}}_{s,j,k}$.

### **Assumption 1**

In the reference configuration, boundary nodes of the polytope *j* are all distributed on the surface of a unit sphere centered at the origin of the *T*–*D*–*R* space. Reference configuration of every polytope $j\in {\mathcal {C}}$ is shown in Fig. 1a.

### *Remark 3*

Although polytope $j\in {\mathcal {C}}$ encloses all data points defined by set ${\mathcal {F}}_j$, $${\mathbf {x}}_{s,j,0}=\begin{bmatrix} T_{s,j,0}/100&D_{s,j,0}&R_{s,j,0}/100 \end{bmatrix} ^T\in {\mathbb {R}}^{3\times 1}$$ and ${\mathbf {x}}_{s,j,0}\in {\mathbb {R}}^{3\times 1}$ do not necessarily assign positions of a data paint belonging to set ${\mathcal {F}}_j$. In other words, ${\mathbf {x}}_{s,j,0}\in {\mathbb {R}}^{3\times 1}$ and ${\mathbf {x}}_{s,j,0}\in {\mathbb {R}}^{3\times 1}$ can represent any arbitrary point inside the tetrahedron $s\in {\mathcal {S}}_j$ that is transformed under homogeneous transformation (25).

### Jacobian matrix ${\mathbf {Q}}_{s,j}$ and displacement vector ${\mathbf {f}}_{s,j}$

Let ${\mathcal {B}}_{s,j}=\left\{ s_1,s_2,s_3\right\}$ and ${\mathcal {I}}_j=\left\{ jN\right\}$ define index numbers of tetrahedron $s\in {\mathcal {S}}_j$ in polytope $j\in {\mathcal {C}}$. For every $s\in {\mathcal {S}}_{j}$ and $j\in {\mathcal {C}}$, positions of vertices of tetrahedron $s\in {\mathcal {S}}_j$ satisfy Eq. (25), thus we can write

26a $$\begin{aligned} \bar{{\mathbf {r}}}_{s_k,g}(t)= & {} {\mathbf {Q}}_{s,j}(k) \bar{{\mathbf {r}}}_{s_k,g,0}+ {\mathbf {f}}_{s,j}(k),\qquad k=1,2,3,~s_k\in {\mathcal {B}}_{s,j},~j\in {\mathcal {C}} \end{aligned}$$

26b $$\begin{aligned} \bar{{\mathbf {r}}}_{jN,g}(t)= & {} {\mathbf {Q}}_{s,j}(k) \bar{{\mathbf {r}}}_{jN,g,0}+ {\mathbf {f}}_{s,j}(k). \end{aligned}$$

Per Assumption 1, $\bar{{\mathbf {r}}}_{jN,g,0}=\mathbb {0}_{3\times 1}$ and

27 $$\begin{aligned} \bar{{\mathbf {r}}}_{s_h,g,0}=\hat{{\mathbf {n}}}_{s_h,j,c},\qquad h=1,2,3,~s_h\in {\mathcal {S}}_j \end{aligned}$$

where the unit vector $\hat{{\mathbf {n}}}_{s_h,j,c}$ is defined by Eq. (8). Elements of matrix ${\mathbf {Q}}_{s,j}(k)$ and vector ${\mathbf {f}}_{s,j}(k)$ are then obtained as follows^21^:

28 $$\begin{aligned} \begin{bmatrix} {\text {vec}}\left( {\mathbf {Q}}_{s,j}(k)\right) \\ {\mathbf {f}}_{s,j}(k)\\ \end{bmatrix} =\begin{bmatrix} {\mathbf {I}}_{3}\otimes {\mathbf {L}}_0&{\mathbf {I}}_3\otimes {\mathbf {1}}_{4\times 1} \end{bmatrix} ^{-1} {\mathbf {p}}(k) \end{aligned}$$

where “${\text {vec}}$” is the matrix vectorization symbol,

$$\begin{aligned} & {\text{vec}}\left( {{\mathbf{Q}}_{{s,j}} (k)} \right) = \left[ {\begin{array}{*{20}l} {Q_{{1,1,s,j}} } \hfill & \cdots \hfill & {Q_{{1,3,s,j}} } \hfill & \cdots \hfill & {Q_{{3,3,s,j}} } \hfill \\ \end{array} } \right]^{T} \in \mathbb{R}^{{9 \times 1}} \\ & {\mathbf{p}}(k) = {\text{vec}}\left( {\left[ {\begin{array}{*{20}l} {{\mathbf{\bar{r}}}_{{s,1,g}} (k)} \hfill & {{\mathbf{\bar{r}}}_{{s,2,g}} (k)} \hfill & {{\mathbf{\bar{r}}}_{{s,3,g}} (k)} \hfill & {{\mathbf{\bar{r}}}_{{jN,g}} (k)} \hfill \\ \end{array} } \right]^{T} } \right) \in \mathbb{R}^{{12 \times 1}} \\ & {\mathbf{L}}_{0} = \left[ {\begin{array}{*{20}l} {{\mathbf{\hat{n}}}_{{s,1,j,c}} } \hfill & {{\mathbf{\hat{n}}}_{{s,2,j,c}} } \hfill & {{\mathbf{\hat{n}}}_{{s,3,j,c}} } \hfill & {{\mathbf{0}}_{{3 \times 1}} } \hfill \\ \end{array} } \right]^{T} \in \mathbb{R}^{{4 \times 3}} . \\ \end{aligned}$$

### Eigen-decomposition of pandemic evolution

We can use polar decomposition to express Jacobian matrix ${\mathbf {Q}}_{s,j}\left( k\right)$ by

29 $$\begin{aligned} {\mathbf {Q}}_{s,j}\left( k\right) ={\mathbf {R}}_{s,j}(k){\mathbf {U}}_{s,j}(k),\qquad j\in {\mathcal {C}},~s\in {\mathcal {S}}_j, \end{aligned}$$

where ${\mathbf {R}}_{s,j}(k)$ is an orthogonal (rotation) matrix and ${\mathbf {U}}_{s,j}(k)$ is positive semi-definite at every discrete time *k*. Because every polytope $j\in {\mathcal {C}}$ transforms without rotation, $\hat{{\mathbf {e}}}_1=\hat{{\mathbf {c}}}_{1,j}$, $\hat{{\mathbf {e}}}_2=\hat{{\mathbf {c}}}_{2,j}$, and $\hat{{\mathbf {e}}}_3=\hat{{\mathbf {c}}}_{3,j}$ for every cluster $j\in {\mathcal {C}}$, ${\mathbf {R}}\left( k\right) ={\mathbf {I}}_3$ at every day *k*, and positive semi-definite matrix ${\mathbf {Q}}_{s,j}\left( k\right) ={\mathbf {U}}_{s,j}(k)$ assigns the linear deformation of tetrahedron $s\in {\mathcal {S}}_j$ in polytope $j\in {\mathcal {C}}$.

**Figure 3:**
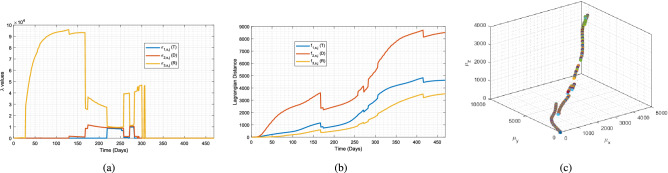
A two-cluster setting, $j\in {\mathcal {C}}$ and $m=|{\mathcal {C}}|=2$ (**a**) Eigenvalues ${\sigma }_{1,s,j}$, ${\sigma }_{2,s,j}$, and ${\sigma }_{3,s,j}$ of matrix $Q_{s,j}$ calculated for node 210. For clustering, the Algorithm 1 is used. (**b**) Distances of node 210 calculated for cluster $j=$“2” (Eq. 24). (**c**) Position of the center of cluster “1” in the T–D–R space, calculated using the Algorithm 1. Note that j=1 and ${\mu }_x$, ${\mu }_y$, and ${\mu }_z$ stand for ${\mu }_{x,1}$,${\mu }_{y,1}$, and ${\mu }_{z,1}$, respectively.

**Figure 4:**
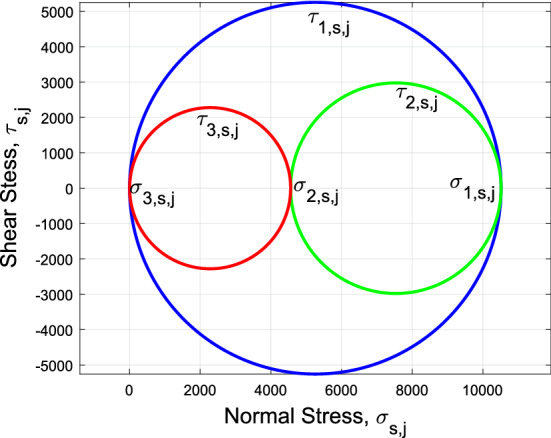
Sample Mohr’s circle for node $S_j$ = 80 and for day $k=$ 220 and cluster j=1 for $j\in {\mathcal {C}}$ and $m=2$.

The eigenvalues of matrix ${\mathbf {Q}}_{s,j}$ are called the **principal values** of ${\mathbf {Q}}_{s,j}$, and are denoted by $\sigma _{1,s,j}$, $\sigma _{2,s,j}$, and $\sigma _{3,s,j}$. In constructing the Mohr’s circle (shown in Fig. 4), $\sigma _{1,s,j}$, $\sigma _{2,s,j}$, and $\sigma _{3,s,j}$ are sorted such that:

30 $$\begin{aligned} 0\le \sigma _{3,s,j} \le \sigma _{2,s,j} \le \sigma _{1,s,j} \end{aligned}$$

Because matrix ${\mathbf {Q}}_{s,j}$ is only time varying, the principal values $\sigma _{3,s,j}$, $\sigma _{2,s,j}$, and $\sigma _{1,s,j}$ are spatially-invariant at every point of tetrahedron $s\in {\mathcal {S}}_j$. Given principal values of tetrahedron $s\in {\mathcal {S}}_j$, we define the following shear stress terms to analyze deformation the pandemic continuum:

$$\begin{aligned} {\tau }_{1,s,j}(k) = (\sigma _{1,s,j} - \sigma _{3,s,j})/2,\qquad {\tau }_{2,s,j}(k) = (\sigma _{1,s,j} - \sigma _{2,s,j})/2,\qquad {\text {and}}\qquad {\tau }_{3,s,j}(k) = (\sigma _{2,s,j} - \sigma _{3,s,j})/2. \end{aligned}$$

Principal and shear stress values can be graphically represented using the Mohr’s circle as shown in Fig. 4 .

#### *Remark 4*

Boundary nodes of tetrahedron $s\in {\mathcal {S}}_j$ are called *active nodes* and tetrahedron $s\in {\mathcal {S}}_j$ is called an *active tetrahedron*, if the volume of tetrahedron $s\in {\mathcal {S}}_j$ is nonzero. Therefore, $\sigma _{3,s,j}>0$, if ${\mathcal {B}}_{s,j}$ defines three active nodes on the boundary of polytope $j\in {\mathcal {C}}$.

Since there are only 52 data points, we chose the number of clusters to be $m=2$ ($j=1,2$), as adding more clusters bears the risk of having empty clusters for some days. For the clustering algorithm, we chose K-means clustering, as a suitable learning algorithm for the T–D–R space. In other words, the purpose of clustering is to obtain two continuum bodies with particles representing the US States and the United States, rather than a single body with heterogeneous particles (data points). By making sure the continuum body is heterogeneous, then we are able to apply continuum mechanics principles to study the growth of the pandemic polytopes. For all cases, the discretization of the *T*–*D*–*R* space is performed using $N=756$ tetrahedral mesh, created by $p=15$ and $q=27$ horizontal and vertical discretized points, in which the polytopes are allowed to elongate and deform without rotation. In Fig. 3a , the eigenvalues ${\sigma }_{1,s,j}$, ${\sigma }_{2,s,j}$, and ${\sigma }_{3,s,j}$ of matrix $Q_{s,j}$, calculated for node 210 are shown. In Fig. 3b the distances of node 210 are calculated for cluster $j=$“2” (Eq. 24). In Fig. 3c , the position of the center of cluster “1” in the T–D–R space, using the Algorithm 1, is shown.

## Growth analysis and pandemic stages

In this section, we establish a growth criteria for the spread of COVID-19 in the United States over 469 days (from March 12, 2020 to June 28, 2021), where the number of infected cases (T), deaths (D), and recoveries (R) are obtained from^22^, which helps us obtain the *T*–*D*–*R* time series data associated with every US State and District of Colombia and at each day of the pandemic. Based on the mathematical foundation discussed in “Eigen-decomposition of pandemic evolution”, eigen-decomposition of the pandemic evolution is performed and principal values of the active tetrahedrons are obtained and plotted in Fig. 6.

**Figure 5:**
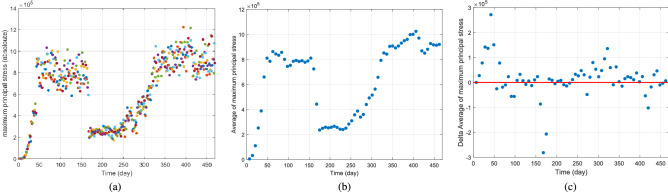
Establishing the growth rate criteria: (**a**) Daily time-series related to cluster $j\in {\mathcal {C}}$ and $m=|{\mathcal {C}}|=2$ of maximum values of principal stresses of $\sigma _{1,s,j}$, $\sigma _{2,s,j}$, and $\sigma _{3,s,j}$ for every active node. (**b**) Filtered signal with an overlapping window with a length of 2 weeks ( 1 week overlap). (**c**) The point-to-point difference in the values of the signal in (**bb**) is defined as the metric for pandemic growth.

Mohr’s circle method, shown in Fig. 4, is used to obtain the maximal stress values of each cluster, for each day. Mohr’s circle is a graphical representation of the Cauchy stress tensor, and helps obtain the principal stress values in the principal planes of a continuum body. As can be seen from Fig. 5a, the daily time-series related to cluster j ($j\in {\mathcal {C}}$) of maximum values of principal stresses of $\sigma _{1,s,j}$, $\sigma _{2,s,j}$, and $\sigma _{3,s,j}$ for every active node is a very noisy signal. Thus, using signal processing methods is needed.

### Signal processing

An overlapping window with a length of two weeks (with 1 week overlap) is used to calculate the average value of the captured data of each window. The reason we chose an overlapping window of length 2 weeks was that it is understood that the incubation of the infection is more of less 2 weeks. Choosing the window to be overlapping keeps the windows of data sustain any temporal phenomenon in the time-series data, that would have been lost using a non-overlapping window. As can be seen from Fig. 5b, the filtered signal, which is the average of data points in the windows from Fig. 5a, is less noisy and hence, better for establishing the growth rate. We define the growth rate to be the point-to-point difference in the values of the signal in Fig. 5b, such that for any day that its signal in Fig. 5c is positive, the pandemic is growing, and for any day that its signal in Fig. 5c is negative, the pandemic is shrinking. A red line is depicted in Fig. 5c for the ease of illustration as well.

The magnitude of the signal values in Fig. 5c determines the rate of growth or shrinkage. In other words, for two “positive” days, the pandemic had a greater growth rate for the day with larger value in Fig. 5c.

### Stages of the pandemic

**Figure 6:**
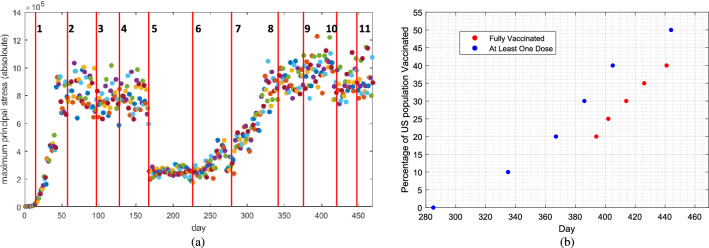
(**a**) Daily time-series related to cluster $j\in {\mathcal {C}}$ and $m=|{\mathcal {C}}|=2$ of maximum values of principal stresses of $\sigma _{1,s,j}$, $\sigma _{2,s,j}$, and $\sigma _{3,s,j}$ for every active node. The vertical lines (from 1 to 11) represent the time of dynamics change in the pandemic evolution. (**b**) Percentage of US population Vaccinated based on time (days). Both the “Fully Vaccinated” and “At Least One Dose” are plotted. The start day of the vaccination is December 21 2020 (corresponding to day 285 of the TDR data). Data is from^23,24^.

In addition to defining the growth criteria, a manual process of selecting “important” dates of the pandemic was carried out. Visually, one can observe distinct days of the time-series (11 days), in which the maximum principal values drastically changed. These days are marked with vertical, red lines in Fig. 6. The working hypothesis in this paper is that each of the 11 lines is caused by (or is correlated with) an State-wide executive order and/or a milestone in population vaccination, in the US. In later sections, we study which executive orders are best “matching” with the 11 event marks depicted in Fig. 6.

In general, there are five different growth stages of any pandemic: lagging (beginning of the outbreak), exponential (rapid growth), deceleration (growth decay), stationary (near zero growth), and linear growth (constant growth above zero)^17^. Looking at Fig. 6a, we can identify the region between y axis and line “ **1**” to include the “lagging” (from day 0 to around 20), the region between line “ **1**” and line “ **2**” to include “exponential growth” (day 20 to 60), the region between line “ **2**” and line “ **3**” to include “growth decay”, while the region between line “ **6**” and line “ **7**” to include can be associated with “linear growth”. Regions between line “ **4**” and line “ **5**” and between line “ **5**” and line “ **6**” exhibit “stationary growth”. The only region that exhibits a negative slope trend is region between line “ **8**” and line “ **9**”.

## Discussion

In this section, we discuss: (1) State-wide orders and vaccination milestones, (2) Define “Net Actions” and investigate the correlation between “Net Actions” and maximum principal stress values, and (3) make suggestions to the Governments and people.

### State-wide orders and vaccination milestones

In the US, a State Governor is authorized to declare a State of Emergency (SOM), in addition to other State-wide orders, within his or her jurisdiction, based on the State’s constitution. These State orders are of high variety, and include travel, education, medical system, entertainments, and business, to name a few^20^. During the COVID19 pandemic in 2020 and 2021 in the US, there was a variability on the dates in which each State Governor declared State-wide orders emergency. We examined a number of different orders among all the States to find the most effective orders in countering the pandemic growth, as discussed below.

**Figure 7:**
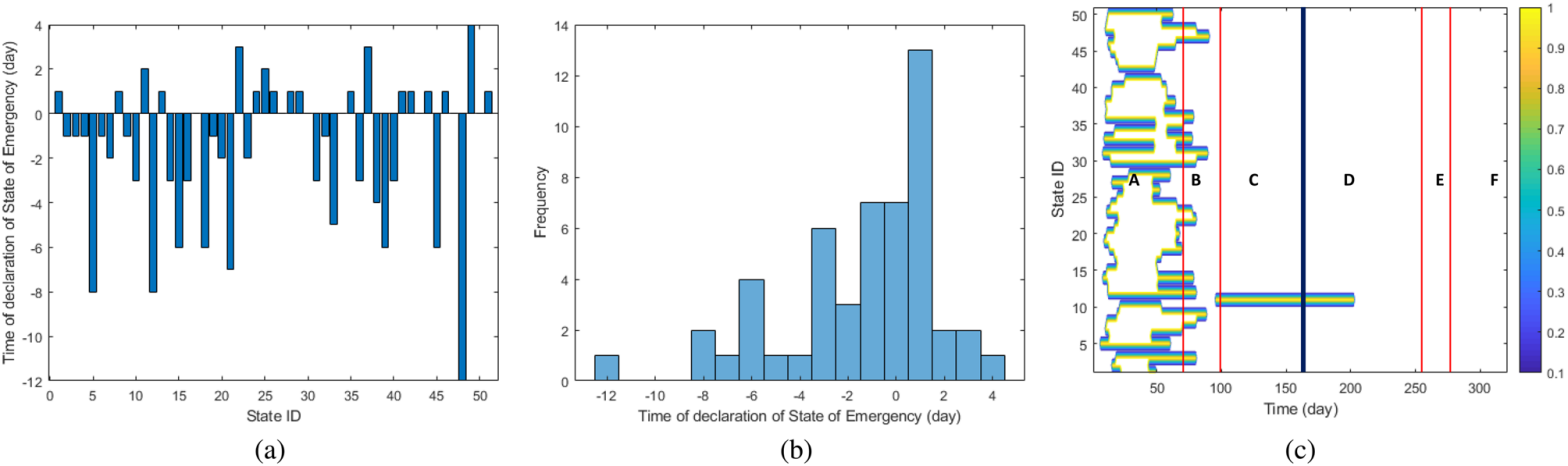
(**a**) The timing of announcement of SOM by each State. The IDs associated with each US State is presented in Table 1. (**b**) The histogram of the timing of announcement of SOM by each State. The histogram almost follows a bell-shape trend, suggesting a normal distribution of SOM announcement timing in the US. (**c**) The timing of announcement of “shelter at place” by each State.The IDs associated with each US State is presented in Table 1.

As can be seem in Fig. 7a, all of the US States declared SOM in a 2-week period, so we can consider the action of US States to be uniform in this regard. The timing of such announcements follow a normal distribution, as can be seen in Fig. 7b, hinting that the Governments’ behaviour in this respect were more or less synchronized. Thus, the pandemic growth is not well-correlated with this order, except for the initial stages of the outbreak.

The “Shelter At Place” executive order is a ordered to significantly reduce social interaction and therefore, the spread of the disease. Previous studies report that the most effective Statewide order to minimize virus spread is shelter at place^20^. As can be seen in Fig. 7c, the timings of announcement of shelter in place amongst States are almost overlapping, even though there are some variability here. The average duration of shelter in place order among States is 44.73 days, with a standard deviation of 22.30 days. The States that has the highest duration of shelter at place were Georgia with 107 days, New Jersey with 80 days, Virginia with 73 days, and New York with 68 days.

Different phases were declared (“Phase Declarations”) during the pandemic, corresponding to the growth of COVID-19 in different times^25^. “Phase 1” corresponds to the rapid spread of the virus in which the public health response relies on dramatic mitigation measures, like stay at home orders and social distancing, to slow the spread of the virus. “Phase 2” corresponds to flattening of the spread and the rise in the rate of infection is beginning to slow and stabilize. Hospitalizations and ICU bed usage continue to increase but are flattening “Phase 2”. In “Phase 3” or recovery phase, the rate of infection is stable or declining. In “Phase 4” or revitalization, there is a continued decline in the rate of infection in new COVID-19 cases, and “Phase 5” corresponds to some kind of new normal situation and one could say the US is “restored” as far as COVID-19 pandemic is concerned.

Vaccination in the US started from December 21, 2020 (corresponding to day 285 of the TDR data). Two sources^23,24^ were used to discover percentage of US population’s vaccination percentage, both for “at least one dose’ and “fully vaccinated” cases (remembering that the majority of available vaccines in the US are administered in two doses). The data can be seen in Fig. 6b.

**Figure 8:**
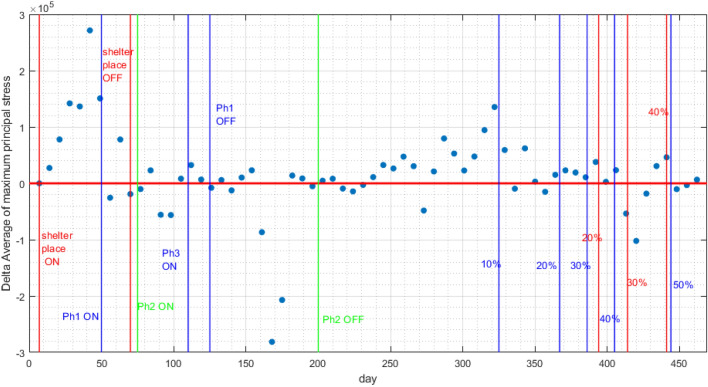
The effect of Governments’ actions on the pandemic growth rate: growth rate is the values of the signal in this figure, such that for any day that signal is positive, the pandemic is growing, and for any day that signal is negative, the pandemic is shrinking.

### Correlation between “net actions” and maximum principal stresses

As mentioned previously, each State has its own timing of declaration of SOM, shelter at place, pandemic phases, and vaccination rate. In order to find a “net action” for the US, we utilize a weighted mean, based on the States’ population, to find a single representative measurement. We found that averaging based on State population density, instead of the population, can be misleading, as for instance, the District of Colombia, has an outlier population density, two orders of magnitude higher than the most of other States^26^. There are other States for which the population density will give artificial weight to States less severely affected by the pandemic.

Let $x_{i}$ and ${{\bar{x}}}$ be State *i* action and the “US net action”, respectively. By “action”, we mean any of the Statewide orders and vaccination percentage milestone. Also, let $w_{i}$ be the ratio of State *i* population to the US population. We have:

31 $$\begin{aligned} {{\bar{x}}}={\frac{\sum \limits _{i=1}^{n}w_{i}x_{i}}{\sum \limits _{i=1}^{n}w_{i}}}={\frac{w_{1}x_{1}+w_{2}x_{2}+\cdots +w_{n}x_{n}}{w_{1}+w_{2}+\cdots +w_{n}}}. \end{aligned}$$

**Figure 9:**
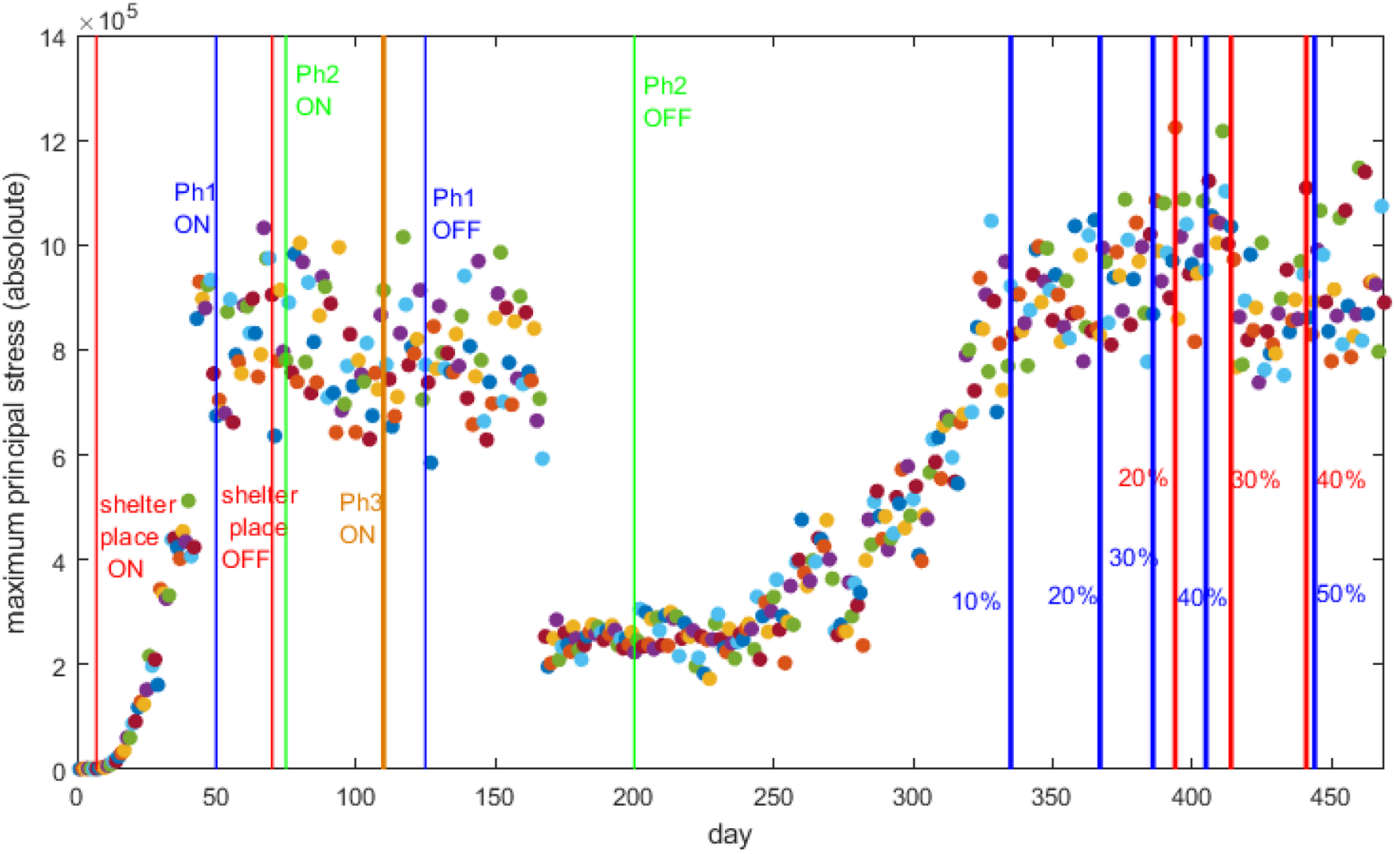
Daily time-series related to cluster $j\in {\mathcal {C}}$ and $m=|{\mathcal {C}}|=2$ of maximum values of principal stresses of $\sigma _{1,s,j}$, $\sigma _{2,s,j}$, and $\sigma _{3,s,j}$ for every active node. The vertical lines represent the events such as start/stop of an executive order, phase declarations, or a milestone in administration of vaccines. We have marked 15 vertical lines, with various color coding. The “shelter in place” ON and OFF “net actions” are marked red, the “phase 1” ON and OFF “net actions” are marked blue, the “phase 2” ON and OFF “net actions” are marked green, the “phase 3” ON “net action” is marked brown, the “At Least One Vaccine Dose” milestones are marked blue, and the “Fully Vaccinated” milestones are marked red.

Looking at Fig. 9, we have marked 15 vertical lines, with various color coding. The “shelter in place” ON and OFF “net actions” are marked red, the “phase 1” ON and OFF “net actions” are marked blue, the “phase 2” ON and OFF “net actions” are marked green, the “phase 3” ON “net action” is marked brown, the “At Least One Vaccine Dose” milestones are marked blue, and the “Fully Vaccinated” milestones are marked red.

Looking at Fig. 6a, we have marked 11 vertical, red lines, without any knowledge/attention of the States “net actions”. When we correlate the events in Fig. 6a with the events in Fig. 9, we can make the following inferences: line “ **1**” is well-correlated with the “shelter in place” ON action, line “ **2**” is well-correlated with the “phase 1” ON action, line “ **3**” is almost correlated with the “phase 3” ON action, line “ **4**” is well-correlated with the “phase 1” OFF action, line “ **6**” happens 25 days after the “phase 2” OFF action, line “ **8**” is almost correlated with the milestone of “10% of US population at least vaccinated by one dose”, line “ **9**” is almost correlated with the milestone of “25% of US population at least vaccinated by one dose”, line “ **10**” is almost correlated with the milestone of “30% of US population fully vaccinated”, and line “ **11**” is almost correlated with the milestone of “50% of US population at least vaccinated by one dose”. There are two dynamic shifts (lines “ **5**” and “ **7**”) which are not correlated with the “net actions” we have studied. These happen on the dates that the centers of clusters experienced a “jump”, as can be seen from Fig. 6a.

### Suggestions to the governments and people

At the time of writing this paper, 619,438 people have lost their lives in the US^22^, but only around $45\%$ of US population have been fully vaccinated^23^. It is imperative to note that the vaccination rates reflects the coordinated Governments’ action/Public’s reaction. In other words, Governments should provide sufficient vaccines while people accept to get vaccinated (vaccine hesitancy is a Public reaction, which is fatal). As can be seen from Fig. 8, vaccination has been very effective for decreasing the growth rate. When 10% of the US population were vaccinated with a single dose at least, around day 325, the pandemic growth starts to decline and then experience shrinking. The largest rate of shrinkage occurred around day 410, when 30% of the US population were fully vaccinated. It is imperative that the Governments push for more aggressive rates of vaccination for this, and future pandemics. Another point is the delay in initial response to the outbreak. For a fact, the initial exponential growth rate of an epidemic significantly determines its severeness^15^. Based on our growth criteria, this exponential growth happened until day 55 of our data. We believe that the Governments should have acted earlier, to avoid letting the exponential growth to continue for almost two months. The times series of shelter at place Statewide orders happened before day 55, and ended before day 100. We believe that shelter at place should have continued for more duration, since when looking at day 250 of our data, one can see another rapid growth of the pandemic. Our suggestion/recommendation to the Governments is that for future pandemics, they act earlier and focus on ways to help people stay at home by providing more financial incentives.

## Conclusion

In this work, we have adopted a new hybrid learning and continuum deformation framework to analyse the COVID-19 pandemic growth in the *T*–*D*–*R* space. The *T*–*D*–*R* space is discretized to create a finite set of nodes and tetrahedrons in which the characteristic polytopes of the training data can evolve in. if the volume of tetrahedron $s\in {\mathcal {S}}_j$ is nonzero, it means that it contains a data point. The maximal principal values of Jacobian matrix ${\mathbf {Q}}_{s,j}$ for every $s\in {\mathcal {S}}_j$ and $j\in {\mathcal {C}}$ is found using the eigen-decomposition technique. Our study has some limitation, for instance it is well-known that number of testing has direct impact on the *T*–*D*–*R* numbers. In the US, only after 11 May 2020 (day 90 of our data set) was that the number of tests reached a reasonably high enough number (more than 375,000 tests are done each day). Future research can look into the dynamics of pandemic growth of each region (between the marked vertical lines) of the pandemic as determined by this work.

## State IDs

The IDs associated with each US State used for clustering is presented in Table 1. The States are ordered alphabetically.

**Table 1:** The IDs associated with each US State used in this paper.

State ID	State	State ID	State	State ID	State	State ID	State
1	Alabama	14	Illinois	27	Nebraska	40	South Carolina
2	Alaska	15	Indiana	28	Nevada	41	South Dakota
3	Arizona	16	Iowa	29	New Hampshire	42	Tennessee
4	Arkansas	17	Kansas	30	New Jersey	43	Texas
5	California	18	Kentucky	31	New Mexico	44	Utah
6	Colorado	19	Louisiana	32	New York	45	Vermont
7	Connecticut	20	Maine	33	North Carolina	46	Virginia
8	Delaware	21	Maryland	34	North Dakota	47	Wisconsin
9	DC	22	Michigan	35	Ohio	48	Washington
10	Florida	23	Minnesota	36	Oklahoma	49	West Virginia
11	Georgia	24	Mississippi	37	Oregon	50	Wisconsin
12	Hawaii	25	Missouri	38	Pennsylvania	51	Wyoming
13	Idaho	26	Montana	39	Rhode Island		
